# An Ethical Puzzle—Two in One

**Published:** 1876-09

**Authors:** 


*An Ethical Puzzle—Two in One.*—The Louisville *Medical News* has been pouring hot-shot into two medical colleges, which, in truth, are not *two* but *one,* for some time. We have watched the fight with interest. It calls the "two in one" college "the phenomenon." The *News* strikes right and left, and with most vigorous blows. It has not left a square inch of cuticle unassailed. Now, this ethical puzzle, this magnificent American enterprise—this "phenomenon" of the *News*—is the offspring of our good, old, ethic-loving friend—Dr. E. S. Gaillard—editor of that ethical blunderbuss, "the largest medical monthly in America."

Our friend—of the blunderbuss—has exercised his powers as an ethical gladiator for ten long years. He has grown gray in ethical ranting. He had a habit of saying during all these years: "Come, see what a beautiful sword I wield in the cause of honor, truth and ethics." This sword, and the hand that wielded it, has been the wonder, as well as the admiration, of the land. There was no one so orthodox; no sword so ready to leap from its scabbard; no ethical warrior so willing to put on his "war-paint" in defence of medical honor wrything under the assaults of Quackdom. Not alone in quackry did our gladiator flourish his encrimsoned blade. He was wont to bathe it in nobler blood. There was no medical man with professional attainments, however lofty--the loftier the better: there was no medical journal in the South, however honest, honorable and true—if it had a large circulation: there was no medical college in our section, however high and pure, but what, if any of these came across his purposes, they were made to feel the cold steel of his ethical blade. In season and out of season: with cause and without cause, this gladiator was always seen, stripped for the fight, in the ethical arena. His name was on every lip. The fraternity sang peans to his ethical fervor all over the land.

This was in the past. *O! tempora! O! mores!* The times change, and gladiators change with them. This marvel of ethical paragons—this wonder of ethical orthodoxy—has fallen, fallen—and become the object of pity to his friends, a bye-word and reproach to his enemies! He now engineers the "phenomenon"—an institution too dark for scrutiny and too hazy for the code. It is one of the freaks of evolution—at least in the evolution of something, however nebulous, from nothing. Its existence is a mystery—its origin a marvel and a wonder It is unique. It stands solitary and alone in its dazzling originality. It is one of those things of which medical philosophy never dreamed. It came, Minerva-like, full grown from the brain of an Olympian Jove—from the mental matrix of that mighty genius who feels no intellectual exhaustion in the editorship of two medical journals; in the Deanship of two medical colleges, to say nothing of the Atlas-like burden of that immense medical enterprise, the Medical Mutual Life Association! Ye gods, pause and behold him! Ye mortals, wonder and adore him!!

All this, while the source of never ceasing wonder might be creditable if it was the mental fruitage of any other mortal than that of the gallant ethical gladiator. But in him, it is a commentary and a puzzle; for he, of all others the firmest, truest friend of the ethics, essays to accomplish his designs by a sublime defiance of its teachings—yea, to carry them into execution over the prostrate form of the law he so often upheld and defended.

The *News* strongly intimates that our *dual* editor and Dean deals in fraud, deception and hypocrisy. It would seem, indeed, that our gladiator had gotten things seriously mixed. He gives the medical world a riddle to solve. He proposes to lay the Delphic oracle in the shade. We cannot follow the tangled skein as he unravels the mysterious process, but we see that, whereas there was "*one*," there is now "*two*," and yet only "*one*!" Mathematics may be a lie; natural philosophy may be false, since it claims that *two* things cannot occupy the same place at the same time, but medical magic and Louisville legerdemain can do all this before our eyes. He proposes to imitate Dame Nature. He makes two, one; and gives birth to young M.D.'s in *nine months*. Now the *News* calls this matrix for the evolution of young M.D.'s—the "Phenomenon." Its offspring must be very *simple* creatures. "*Similia, similibus*" *propagantur*.

Our gladiator, having worked out the puzzle to his satisfaction, *i. e.*, how to make *two* out of *one* and still remain *one*, opens wide the doors of his *two* colleges in the *one* building, with *one* set of trustees and teachers, for aspiring young men agonizing to wear the questionable honor it confers. Now, "the manner of the doing" in order to secure patronage is fully set forth by the naughty *News*. The "phenomenon" wields a large and mighty beneficiary list. It advertises, like every other quack, with this difference only: the quack pays for his advertising, the "phenomenon" dead-beats its way through the press, secular and religious. Its monotonously worded beneficiary notices are published from Maine to Texas, from the Lakes to the Gulf, and the press is "dead-beat" out of its pay under the idea of advertising a charity. This is what that spunkey *News* avers. Every editor, yea every public man or woman of every State, has been notified by circular that he, she or it, is entitled to *name* a beneficiary, and graciously informed that $80 will be deducted from the regular fees. This is the beneficiary dodge. The beneficiary reaches Louisville, and finds, to his dismay, that he has as much to pay as in almost any Southern college; more in fact than in any other except the University of Louisiana. That is the working of the beneficiary dodge. The *News*, watching the running of the machine on the spot, seems to regard this indigenous American conception a humbug and a fraud.

Now, it may not be in "good taste" in us to speak second hand, but the "phenomenon" exists, and if the *News* is correct the average professional mind will not fail to diagnose the condition of things at Louisville, and define the ethical status of the *dual* editor and Dean, as well as his gorgeous "phenomenon" —the medical puzzle and wonder of not "one" but "two" continents.

